# Anatomy of teleost fish immune structures and organs

**DOI:** 10.1007/s00251-020-01196-0

**Published:** 2021-01-11

**Authors:** Håvard Bjørgen, Erling Olaf Koppang

**Affiliations:** Section of Anatomy, The Faculty of Veterinary Medicine, Norwegian University of Life Sciences, Ullevålsveien 72, Oslo, Norway

**Keywords:** Bursa, Fish, Histology, ILT, Immune organ, Kidney, Lymphoid tissue, Morphology, Spleen, Thymus

## Abstract

The function of a tissue is determined by its construction and cellular composition. The action of different genes can thus only be understood properly when seen in the context of the environment in which they are expressed and function. We now experience a renaissance in morphological research in fish, not only because, surprisingly enough, large structures have remained un-described until recently, but also because improved methods for studying morphological characteristics in combination with expression analysis are at hand. In this review, we address anatomical features of teleost immune tissues. There are approximately 30,000 known teleost fish species and only a minor portion of them have been studied. We aim our review at the Atlantic salmon (*Salmo salar*) and other salmonids, but when applicable, we also present information from other species. Our focus is the anatomy of the kidney, thymus, spleen, the interbranchial lymphoid tissue (ILT), the newly discovered salmonid cloacal bursa and the naso-pharynx associated lymphoid tissue (NALT).

## Introduction

In 2000, Zapata and Amemiya (2000) published a review paper addressing the ontogeny of immune structures in vertebrates. Anatomy is one of the most ancient disciplines in medicine, and the old saying goes: “*Anatomia medicinae fundamentum est*”. Therefore, by all standards, one would have thought that major lymphoid tissues in fish should have been discovered and described by 2000. However, this was not the case. Since then, large lymphoid structures, previously un-recognised, have been identified in fish, and in addition, continuous research efforts have provided complementary information regarding the tissues already known.

What is a lymphoid organ? An organ, lymphoid or not, may be defined as a “somewhat independent body part that performs a specific function or functions” (Studdert et al. 2012). Organs are again made up of tissues and a good and useful definition of a tissue is “a group or layer of similarly specialised cells that together perform certain special functions” (Studdert et al. 2012). Most tissues contain lymphoid cells, but that does not necessarily make them lymphoid. However, if the tissues predominantly contain lymphoid cells, they are frequently arranged in lymphoid organs. The precise definitions are in other words not clear-cut. Following the terminology presented above, it is not easy to define a lymphoid tissue if it cannot be visualised as an aggregate of lymphoid cells, e.g. the mammalian Peyer’s patches, which although being an integrated part of the abdominal wall also are defined as secondary lymphoid organs (Pabst et al. 2007). To refer to diffuse lymphoid tissue, which is common in piscine immune research, seems alien according to traditional anatomical terminology. In our opinion, the term “lymphoid tissue” outside a well-defined localization predominantly consisting of lymphoid cells is confusing and should be avoided. For instance, it is common to describe lymphoid cells in the intestines of fish as gut-associated lymphoid tissue and that fish have a “diffuse lymphoid tissue” at this location. To have a “diffuse tissue” is contradictory with respect to the given definition of a tissue. The intestinal lymphocytes are indeed part of a tissue (the intestinal mucosa), but they do not form a tissue in its own right. A more precise terminology describing the existence of leukocytes in the fish intestines would be to report what is objectively observable, namely intestinal mucosa infiltrated with in most cases scattered leukocytes. The mucosa is divided into two effector compartments, i.e. the epithelium and the lamina propria, which are separated by the basal membrane. The basal membrane is permeable and allows traffic of leukocytes from one compartment to the other. This traffic occurs through fenestrations of the basal membrane which are more prominent at sites with mucosa-associated lymphoid organs (MALTs) (Takeuchi and Gonda 2004). In mammalian anatomy, there is a clear distinction between the ordinary mucosal surface and that containing MALT structures, which only are found at certain locations. As emphasised by Smith et al. (2013), these locations are not to be confused. So, although well-established in the piscine immunology community, we therefore argue for not using a terminology implying that a “diffuse tissue” is a tissue. In the following, we will use the term “lymphoid tissue” as an area where predominantly lymphoid cells are observed within defined structural boundaries, and such sites are normally confined to lymphoid organs. This review will therefore not cover mucosa-associated lymphoid cells which recently have been reviewed separately (Salinas and Miller 2015; Bjørgen et al. 2020), but with the exception of discrete lymphoid structures.

In our opinion, morphological studies of the fish immune systems seem somewhat forgotten in the age of genomics and proteomics. Researchers dealing with zebrafish have a tendency of regarding this fish as a swimming human, or at the best, as a swimming human embryo, with no biological rights in its own. But teleost fish evolved millions of years ago (Zapata and Amemiya 2000), and they are finely tuned to their lifestyle and habitat. They have not simply been frozen in their evolution at a given developmental stage but have rather over these years developed their own solutions. This fact seems frequently to have been neglected by researches only regarding fish as model organisms. A stunning example of the discrepancy is the debate, or even lack of debate, regarding the existence of a lymphatic vessel system in fish. It has long been established and proven that fish have a secondary vascular system. This secondary vascular system may appear as lymphatic vessels, but it is not. However, some researchers seem to ignore this fact and have frequently been reporting about the lymphatics of fish. This is especially so for researchers using zebra fish as model organisms. Apparently, their swimming human has no solutions by its own. This negligence of available information prompted Vogel (2010) to publish an excellent comment regarding this topic. In a striking comment, he stated that “Given that all actinopterygian fish studied thoroughly have been shown to have a secondary vascular system, it would be extremely surprising if zebrafish were the only exception.” Other researchers have taken this information into account when presenting their results and arguing for the existence of lymphatic vessels in teleost fish (Hellberg et al. 2013). And that may well be so. Based on available information, it is a fair assumption that teleost fish possess a secondary vascular system which by no means is equivalent to a lymphatic vessel system, but that fish in addition may have lymphatic vessels, most probably related to the intestines (Hellberg et al. 2013). This is just one example of many where scarce anatomical information has a great impact on our perception on the function of the immune system, and where additional morphological studies are highly warranted.

**Fig. 1:**
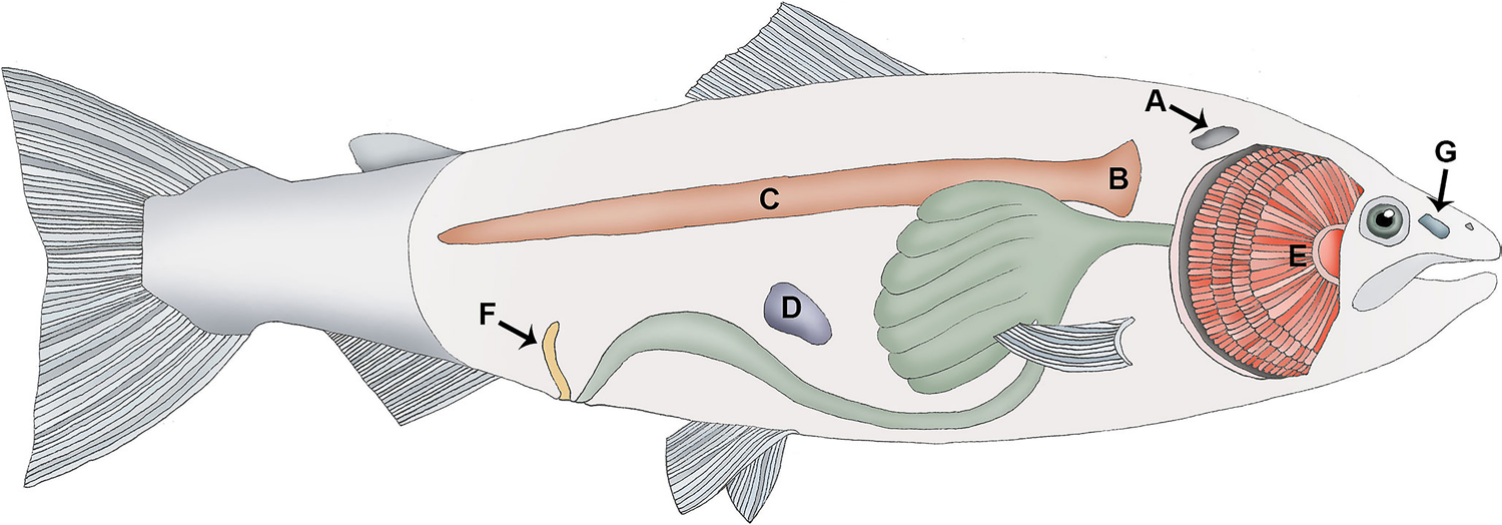
Schematic topography of immune organs in Atlantic salmon. **A** Thymus, **B** head kidney, **C** trunk kidney, **D** spleen, **E** gills with the interbranchial lymphoid tissue (ILT), **F** salmonid bursa, **G** olfactory organ with the nasopharynx-associated lymphoid tissue (NALT)

Our scope is to give a broad overview of teleost immune tissues in an anatomical context focusing on salmonids (Fig. 1). Other tissues not mentioned in this review contain varying amounts of immune cells. In the salmon industry, where fish is intraperitoneally vaccinated, chronic inflammatory intra-abdominal changes have been frequently reported. The reader is referred to special literature with respect to these issues. The work by Zapata and Amemiya (2000) serves as a comprehensive fundament for our review. Other excellent reviews are also available, for instance Press and Evensen (1999). These works cover several fundamental aspects related to kidney, thymus and spleen. With respect to the nasopharynx-associated lymphoid tissue, a recent and excellent review has been published (Das and Salinas 2020), and this tissue will therefore only briefly be presented here. However, the recently discovered immune structures interbranchial lymphoid tissue (ILT) (Haugarvoll et al. 2008) and the salmonid bursa (Løken et al. 2020) need to be presented as lymphatic structures in the context of other fish immune organs and will therefore be of focus in this review.

## Thymus

The thymus of teleosts is a paired organ placed in a dorsal projection in the epithelium of the opercular cavity. It is covered by the pharyngeal epithelium which forms a barrier towards the external milieu (Castillo et al. 1998). The organ consists mainly of reticulated epithelial cells forming niches in which T cells are embedded (Chilmonczyk 1983; Castillo et al. 1990). The organogenesis involves thymic bud formation from pharyngeal pouches (Chilmonczyk 1992). Here, lymphoblasts seed the epithelium and start proliferating. The origin of these cells is not established; they have been suggested to have migrated from both kidney tissue and from the yolk sac (Zapata and Amemiya 2000; Chilmonczyk 1992; Bowden et al. 2005). The innervation of the thymus is sympathetic and nervous tissue may origin from the fourth or fifth sympathetic ganglion in those few fish species which have been investigated (Chilmonczyk 1992). The blood supply originates from vasculature penetrating the organ from the subepithelial connective layer and subsequently branching out associated with thymic septa. Transverse sections of such septae may easily be interpreted as Hassall’s corpuscles, and their presence in teleost thymus is therefore disputed (Chilmonczyk 1992). Zapata and Amemiya (2000) noted in their review that in the phylogenesis, such corpuscles first appear in birds. From the septae, the blood vessels branch out and divide in capillaries surrounded by a discontinuous layer of epithelial cells (Chilmonczyk 1992).

In vertebrate evolution, the thymus appears to be the first primary lymphoid tissue to have been evolved. Its role is to make a niche harbouring developing T cells. The pharyngeal region including the gill arches seems to have been a prime location for the development of lymphoid structures (Matsunaga and Rahman 2001; Varga et al. 2008). In cyclostomata (like lampreys), tissue with some thymic properties has been identified in the gill tips (Bajoghli et al. 2011). Jawless vertebrates possess variable lymphocyte receptors (VLRs) and thus an adaptive immune response similar to jawed vertebrates (Pancer et al. 2004). However, an immune system based on the adaptive properties of receptors of the immunoglobulin superfamily first emerged in jawed vertebrates (Zapata and Amemiya 2000; Flajnik 2018), and all such animals have thymi, where T cells proliferate and mature.

**Fig. 2:**
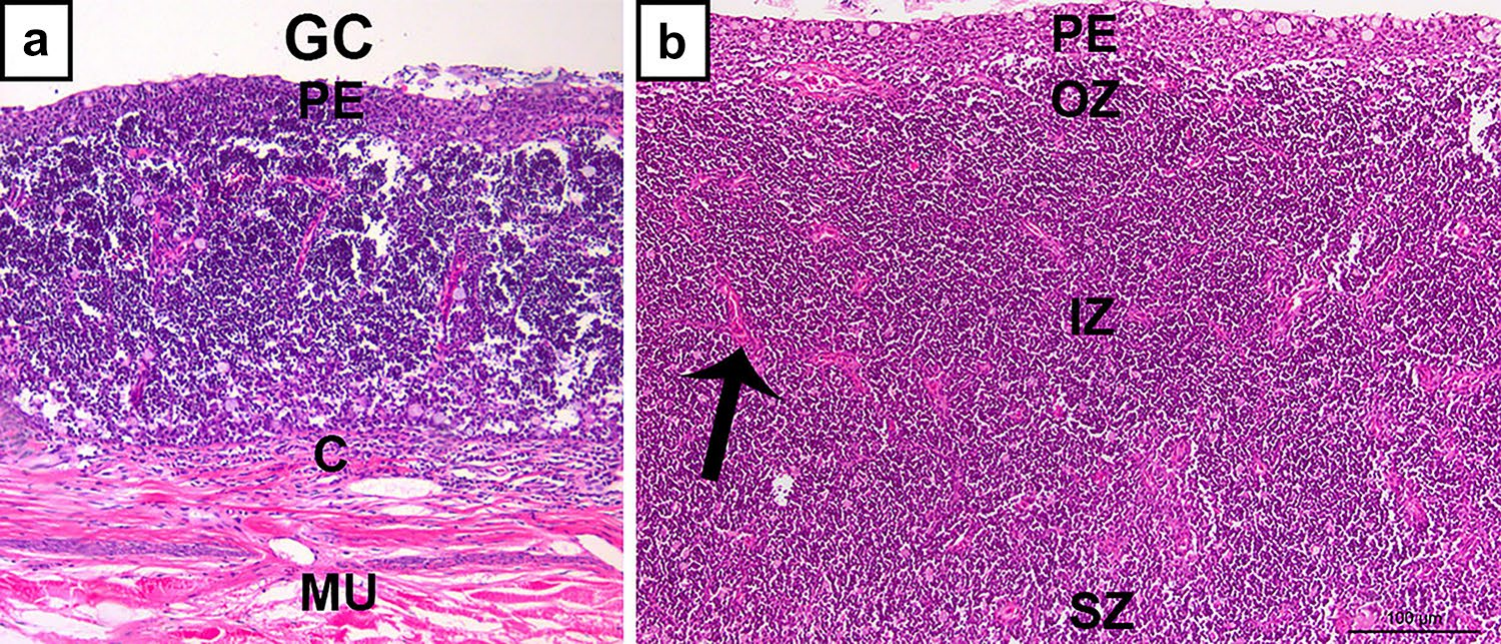
Micrograph of thymus, transverse sections, Atlantic salmon, HE stain. **a** Overview with gill chamber (GC) and underlying musculature (MU). Note the well-developed pharyngeal epithelium (PE) and the capsule (C). **b** Higher magnification shows pharyngeal epithelium (PE), outer zone (OZ), inner zone (IZ), and subcapsular zone (SZ). Arrow indicates a longitudinal section of a septa which might be mistaken for a Hassall’s corpuscle in a transverse section

As noted in their review from 2005, Bowden et al. (2005) showed to a great variation in reported thymus morphology both between fish species and within species. The thymus is a dynamic organ which develops at early age and in most fish species undergoes involution following sexual maturation. Flajnik (2018) noted that thymic organization in gnathostomes (including teleosts) usually follows the classic cortex/medulla organization as known from mammals. This compartmentalization is important, because it provides specialised immunologic niches that are imperative for T cell development. In the cortex, there is proliferation and recombination activity (positive selection) of T cells. In the medulla, negatively selected T cells undergo apoptosis. Surviving and mature T cells may leave the organ through the medullary vasculature. In salmonids, it has been difficult to identify medullary and cortical organization. Rather, the trout thymus may be divided into an outer capsular zone (C) followed by a subcapsular zone (SZ), an inner zone (IZ), an outer zone (OZ) and finally the pharyngeal epithelium (PE) (Fig. 2) (Chilmonczyk 1983,with some modifications from Castillo et al. 1990). So far, a division of a salmonid thymic medulla and cortex, essential for the understanding of the dynamics of the organ, has not been established. This is not so in zebrafish. Here, regionalization of cortex and medulla was established using in situ hybridization for recombination activating gene-1 (RAG-1) (Lam et al. 2002). Such expression is found in T cells confined to the cortex. Similar approaches were used to study the thymus of the common carp (*Cyprinus carpio*) (Huttenhuis et al. 2005) and flounder *Paralichthys olivaceus* (Wang et al. 2014). Different methods have been used to identify the thymic organization of the European sea bass, *Dicentrarchus labrax* (Abelli et al. 1998; Paiola et al. 2017; Picchietti et al. 2015). Even without applying such methods, Mohammad and co-workers (2007) identified a similar distinction in the Australian lungfish, *Neoceratodus forsteri*.

In 2005, Fischer et al. (2005) published a study in rainbow trout, showing that the thymus was the first organ among the lymphoid tissues in which MHC class I protein was observed. Further, these authors applied hitherto novel morphological methods in discovering the sonication of the trout thymus. It was first in 2010 that a description of the normal distribution of T cells in a range of lymphoid and non-lymphoid tissues in a fish species was published (Koppang et al. 2010). Here, T cells were found to be abundant in both the thymus, the interbrancheal lymphoid tissue (ILT) and in the head kidney. Following the generation of antibodies recognizing Atlantic salmon Mhc class II molecules, Koppang et al. (1998) also addressed the distribution of immunopositive cells in a range of salmon tissues. In both studies, the distributional pattern of immunopositive cells within the salmon thymus was observed and described. In rainbow trout (*Oncorhynchus mykiss*), the distribution of thymic Mhc class II positive cells was published in 2005 (Fischer et al. 2005). However, based on these results, it was still difficult to arrive at a conclusion regarding salmon medullary and cortical organization. In a recent study, Bjørgen et al. (2019a) used *in situ* hybridization techniques to address the expression pattern of the cytokine CCL19 in some salmon immune organs. In the mammalian thymus, CCL19 is known to be present in structural cells especially abundant in the junction between the thymic cortex and medulla. In the salmon thymus, positive cells were in particular confined to a layer between the outer zone and inner zone. Clearly, there is a great need for more research within this field. It seems reasonable to assume that studies of RAG expression will be useful for clarifying the matter of thymic organization further in salmonids.

## Kidney

The kidney develops in so-called generations — in birds and mammals, there are three stages or generations of kidney development: the pronephros, the mesonephros and the metanephros. Here, the pronephros and mesonephros undergo atrophy with the resulting metanephros as the permanent kidney. The kidney has both exocrine functions as it produces urine, and in addition endocrine functions with hormone production (Hyttel et al. 2009). In fish, there is no formation of the metanephros, but after organogenesis, the pronephros and mesonephros persist together as the cephalic or head kidney and the exocrine or trunk kidney, respectively (Fernandes et al. 2019). In salmon, blood supply to the excretory and other parts of the kidney is provided by *aa. renalis* coming of *aorta dorsalis*, with its afferent arterioles supplying the capillaries of the glomeruli. From the glomeruli, blood drains via efferent arterioles to the sinusoids. The sinusoids are numerous in the kidney interstitium, and in addition to receiving blood from afferent arterioles, they are supplied with venous blood from segmental veins from the lateral and dorsal musculature and the skin, thus constituting a renal portal system. The venous drainage goes through the *vv. cardinales caudales*. Noteworthy, *v. cardinals caudales sinister* is often shorter and of lower diameter and in many species rudimental, leaving *v. cardinales caudales dexter* as the primary draining vein of the kidney in most salmonids. This vein receives blood from *v. caudalis* and is thus much thicker, which can be visualised in a macroscopic cross section of the kidney (Harder and Sokoloff 1976).

**Fig. 3:**
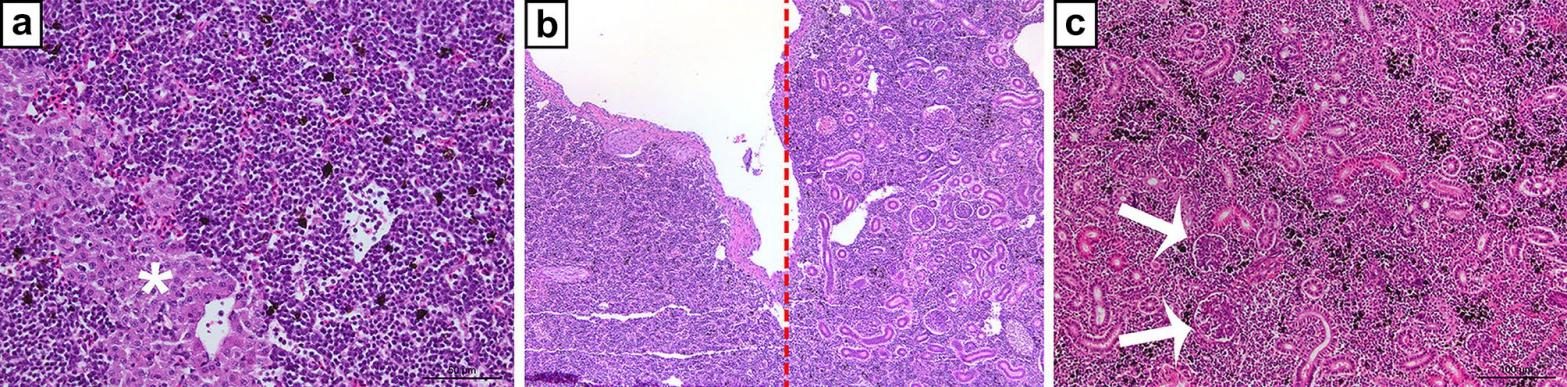
Kidney, Atlantic salmon, HE stain. **a** The head kidney is dominated by hematopoietic interstitial tissue. Note the abundance of melano-macrophages (black). Endocrine tissue (adrenal homolog) is marked with asterisk. **b** The transition between head kidney (HK) and trunk kidney (TK) is marked with a red, dotted line. Note the appearance of glomeruli and tubuli in the trunk kidney. **c** The trunk kidney is dominated by exocrine tissue with nephrons and tubuli. Multiple glomeruli are present in the image (arrows). The interstitium contains hematopoietic tissue and melano-macrophages (black)

In the head kidney, there is haematopoietic tissue, and this tissue is regarded as the teleost bone marrow equivalent due to the structural similarity with bone marrow of higher vertebrates (Fig. 3) (Zapata and Amemiya 2000; Press and Evensen 1999). Functional and transcriptional studies further support this assumption and B cell development has been addressed in several studies (Zwollo et al. 2005; Rombout et al. 2005). In addition, the head kidney has antigen-sampling abilities and antigen retention has been demonstrated in several studies (reviewed in Press and Evensen (1999)). Sinusoidal endothelial cells, macrophages and fibroblastic reticular cells form the actively phagocytic elements (Press and Evensen 1999). It is commonplace in piscine pathological procedures to isolate pathogens causing systemic infections from the head kidney. Further, as is a normal feature of secondary lymphoid organs, immune genes in the head kidney are up-regulated following a variety of antigen challenges. For instance, vaccination has been shown to induce Mhc up-regulation (Koppang et al. 1998a, b). These features have led fish researchers to regard the head kidney as both a primary and secondary lymphoid organ.

The ontogeny of Mhc class I and its receptors in kidney tissue was addressed by both morphological and transcriptional approaches by Fischer et al. (2005). Morphological studies have demonstrated that Mhc class II^+^ cells and T cells are dispersed throughout the kidney (Fischer et al. 2005; Koppang et al. 2010, 2003). Interestingly, there is a maturation gradient of B cells. The head kidney contains mostly proliferating B cell precursors and plasma cells, whereas the trunk kidney contains abundant B cells, some of which are activated, and in addition plasmablasts (Zwollo et al. 2005). Histologically, most Mhc class II^+^ cells in the kidney tissue seem to be macrophages. But in addition to the B and T cells and macrophages, a prominent cell population in the salmonid kidney is melano-macrophages (Agius and Roberts 2003). Previously, they were thought only to accumulate melanin. However, in 2006, Haugarvoll et al. showed that the SHK-1 cell line, which had been classified as a cell line consisting of macrophage-like cells isolated from salmon head kidney, was capable of melanin synthesis (Haugarvoll et al. 2006). Further, the salmon genes for the products of the tyrosinase gene family, which encode enzymes in the melanin synthesis pathway, were cloned and sequenced by Thorsen et al. (2006), showing high expression in head kidney tissue and in the SHK-1 cell line. These results suggest that salmon leukocyte populations are capable of synthesizing melanin, and that is in keeping with the situation in amphibia (Sichel et al. 1997). The rationale for this activity remains un-resolved. In antigen exposure and infection experiments, the melanin-producing capacity of the SHK-1 cells only seemed influenced by temperature, but not by antigen exposure or virus infection (Larsen et al. 2013). Melano-macrophages or melano-macrophage centres are found in several organs of the fish and in chronic inflammatory conditions, but they are especially rich in kidney tissue (Agius and Roberts 2003; Thorsen et al. 2006; Bjørgen et al. 2019b).

Different endocrine cells are present in the kidney tissue. In the head kidney, they include thyroid cells arranged in follicles, which may be located outside or within the tissue dependent on species (Geven and Klaren 2017). Also, the teleost adrenal counterpart is located here, and such tissue may be observed as islands between haematopoietic tissue (Fig. 3) (Di Lorenzo et al. 2020). In the trunk kidney, corpuscles of Stannius may be observed as discrete structures containing cells producing stanniocalcin-1 (STC-1), the dominant calcium regulatory hormone of fish (Greenwood et al. 2009). Adrenal gland cells are innervated by the sympathetic nervous system (Gallo and Civinini 2003); in addition, parasympathetic fibres may also be found. In histological sections of kidney tissue, it is therefore quite common to see thick peripheral nerves trans-passing the tissue.

In the trunk kidney, glomeruli and proximal and distal tubuli may be found (Fig. 3). Hematopoietic tissue is dispersed between these structures. As in mammals, the proximal tubuli contain microvilli and may be highly MHC class II positive, just like in mammals (E.O. Koppang, unpublished result). Fish do not possess the loop of Henle rigidly arranged as in higher vertebrates, and therefore, their kidney tissue cannot be divided into a cortex or a medulla. Consequently, no osmotic gradient like in the mammalian or avian metanephros can be established, implying that the ability of the fish kidney to concentrate urine is very limited. On the ventral surface of the kidney tissue, two ducts may be observed. These are the ureters, or embryologically the ducts of Wolff (Demoll and Harder 1964).

## Spleen

**Fig. 4:**
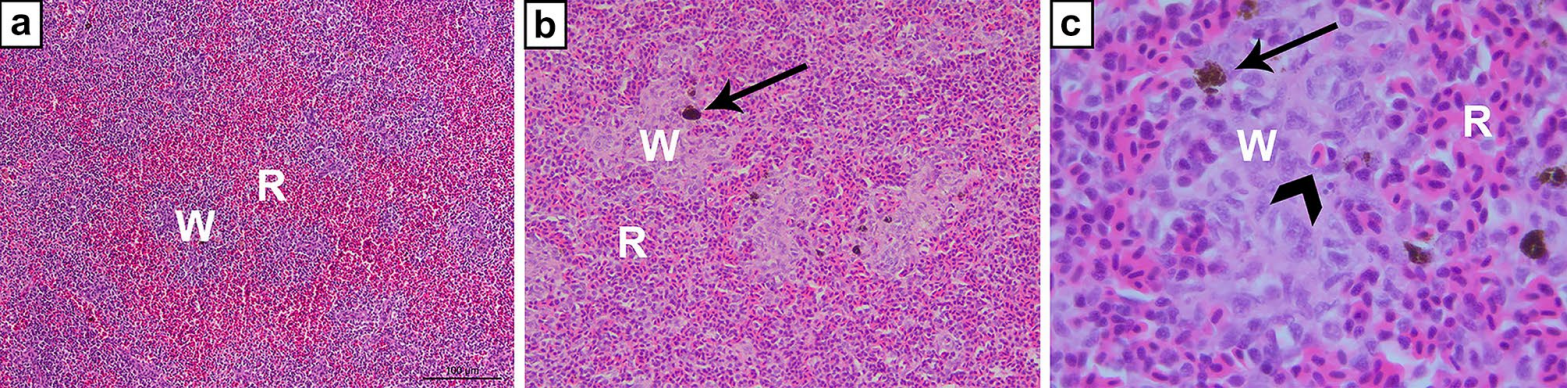
Spleen, Atlantic salmon, HE stain. **a** At low magnification, the histological difference between the white pulp (W) and the red pulp (R) is more prominent than at higher magnification. **b** Higher magnification allows identification of melano-macrophages (arrow, black cells) in the white pulp (W) whereas no such cells are present in the red pulp (R). **c** At high magnification of the white pulp (W), an ellipsoid containing an erythrocyte (arrowhead) can be observed together with scattered melano-macrophages (arrow, black cells)

The spleen is regarded as the primordial secondary lymphoid organ, and almost all gnathostomes possess this organ in which adaptive immune responses are generated (Flajnik 2018). The primordium of the spleen arises from local mesenchyme derived from mesoderm, and a complex vasculature forms within this anlage. Subsequently, there is a development of the white pulp following leukocyte infiltration (Hyttel et al. 2009). The spleen is constructed to filtrate peripheral blood. Visceral arteries supply the organ, whereas the draining splenic veins join the hepatic portal system. The teleost spleen is innervated by sympathetic fibres, and there are no indications of vagal innervation (Fänge and Nilsson 1985). The arteries branch out in the splenic tissue where they terminate in so-called ellipsoids. Their structure varies between different fish species (Fänge and Nilsson 1985), but the construction in trout seems to be representative for most investigated species. Here, the ellipsoids consist of cubical endothelial cells, contrasting the more flattened ellipsoidal endothelial cells in mammals. There are gaps between the endothelial cells, and the underlying basal lamina is disrupted. This allows an effective filtration through the structures. The basal lamina is surrounded by reticular cells and macrophages. Together, these cells and structures form the white pulp (Fig. 4) (Espenes et al. 1995a, b). Mhc class II^+^ cells, most likely macrophages, and T cells are abundantly present in the white pulp (Koppang et al. 2003, 2010). The construction is principally similar compared with the situation in mammals. Filtration occurs through sheets of leucocytes of the white pulp and is finally released in the red pulp which really just consists of peripheral blood surrounding the white pulp (Fig. 4).

In the spleen, fish harbour melano-macrophages that, depending on species, can be arranged either in clusters or more loosely dispersed within the white pulp (Agius and Roberts 2003). It has been thought that such sites serve as “dumping sites” for discarded material of different nature, which has been supported by several studies addressing antigen retention (Agius and Roberts 2003). Genes involved in the melanogenesis pathway have been shown to be expressed in the spleen (Thorsen et al. 2006), suggesting de novo synthesis of melanin in the organ.

A number of different infections can cause enlargement of the spleen (splenomegaly) (Noga 2006), reflecting its function as a secondary lymphoid organ. In principal, splenomegaly can occur as a consequence of proliferation of cells in the white pulp or retention of peripheral blood in the red pulp or both.

## Interbranchial lymphoid tissue

**Fig. 5:**
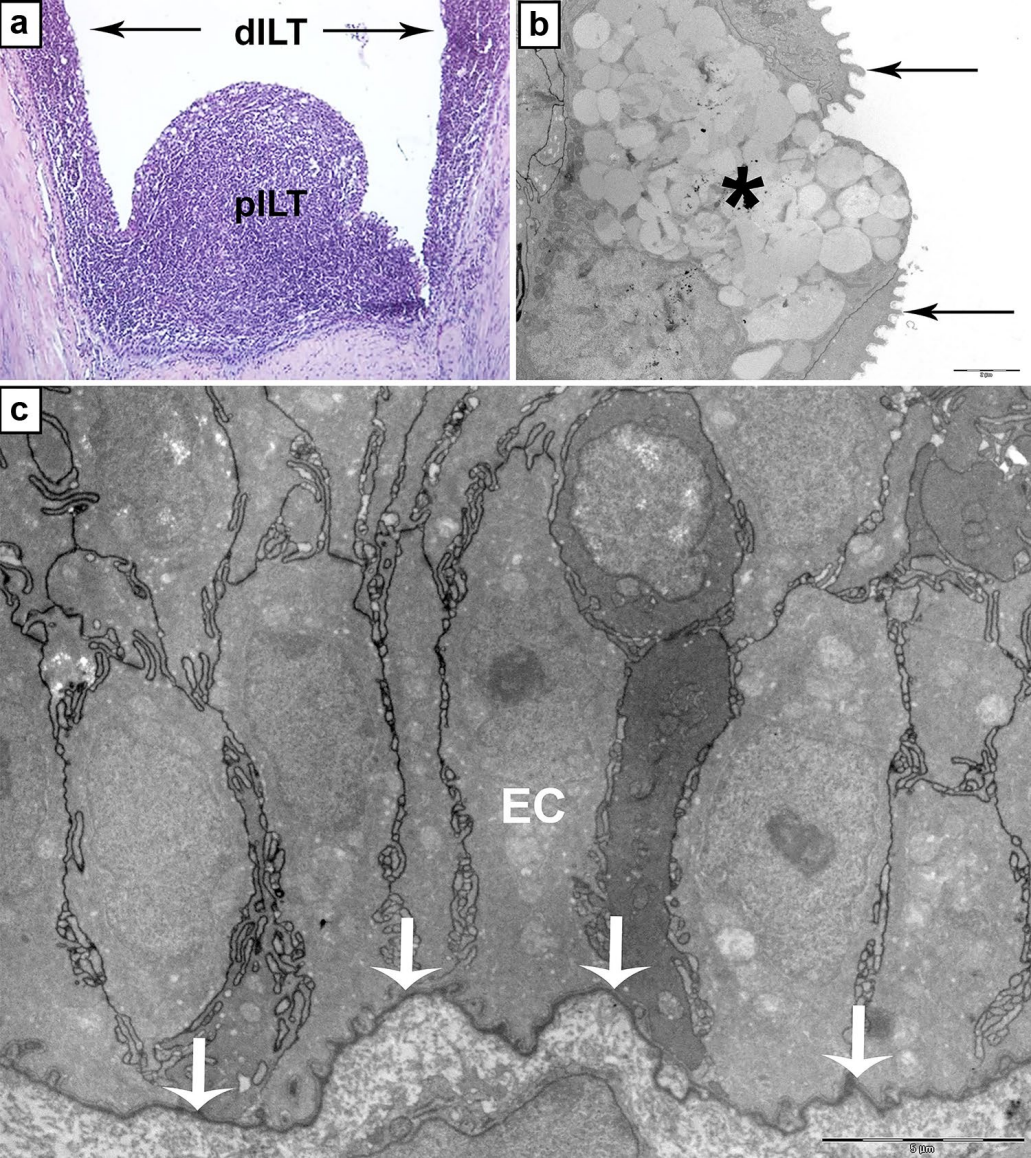
Interbranchial lymphoid tissue (ILT) of Atlantic salmon. **a** Micrograph showing that the ILT is divided into a proximal (pILT) and distal (dILT) compartments consisting of reticular epithelial cells with pockets containing predominantly T lymphocytes. **b** Transmission electron microscopy (TEM) image of the epithelial surface of the pILT showing flattened epithelial cells with microridges (arrows) partly covering a goblet cell (asterisk). **c** TEM image showing the basal membrane (arrows) and epithelial cells (EC) of the pILT in a sexually mature Atlantic salmon. Note the protrusions of the basal membrane interacting with the proximal cell membranes of the epithelial cells of the *stratum basale* and the interdigitating lateral cellular extensions of these cells. This construction enhances the attachment of the pILT to the basal membrane and solidifies the structure. The construction does not seem to facilitate transport over the basal membrane

It has long been known that the fish gills, heavily exposed to the external milieu, have a central role in fish immunology. During ontogenesis, pharyngeal arches give rise to these structures, and their medial epithelium originates from endoderm whereas their lateral epithelium originates from ectoderm (Gillis and Tidswell 2017). In mammals, the juxtaposed pharyngeal endoderm and ectoderm give rise to a range of lymphoid structures including thymus, tonsils and other secondary lymphoid structures commonly known as the Waldeyer’s lymphatic ring (Varga et al. 2008). In fish, tonsils have not been described, but in the salmonid gills, lymphoid tissue known as the interbranchial lymphoid tissue (ILT) (Fig. 5) was first discovered and described about 10 years ago (Haugarvoll et al. 2008). It has later become apparent that such lymphoid aggregates not only are present in salmonids but may also be found in several other teleost species (Rességuier et al. 2020). The ILT is situated between the primary lamellae at the distal extension of the intralamellar septum and was thus named the “interbranchial lymphoid tissue” or “ILT” by Koppang et al. (2010). The ILT consists of lymphoid cells, predominantly T cells, embedded in a meshwork of epithelial cells, but with few and scattered B cells and some strongly Mhc class II^+^ cells (Koppang et al. 2010). This construction has apparent similarities with the thymic outer zone (Bjørgen et al. 2019a). Following its initial discovery, the ILT was subsequently divided into a proximal part (pILT), or the original described lymphoid tissue, and a distal part (dILT), extending along the trailing edges of the primary lamellae (Dalum et al. 2015). In 2020, CCL19 expression was observed in the salmonid ILT (Bjørgen et al. 2019a), arguing for that the ILT should be considered as a lymphoid organ its own right. The ILT of salmonids is strictly intraepithelial and detached from the underlying tissue by a thick basal membrane. Thus, there is no vascularization of this structure, and most probably no innervation (Fig. 5).

The ILT develops precisely at a site of juxtaposed ectoderm and endoderm. At early age, it is not possible to recognise the structure. However, over time, it develops into a substantial size, clearly visible with the naked eye, extending as a ridge along the gill arch and placed between the primary gill filaments (Koppang et al. 2010; Dalum et al. 2016). It is plausible that it develops like the thymus with immigration of T cells through the basal membrane. The epithelium would then start forming niches, analogous with early stages in the organogenesis of the thymus. However, in contrast to the thymus, there is no vascularization of the ILT (Bjørgen et al. 2019a). The structure does not regress in size following sexual maturation but has reached a considerable size in sexually mature salmon (Dalum et al. 2016). Together, these features make the ILT different from any previously described lymphoid tissue in any species.

So far, there is no evidence for recombinatory events within the ILT. However, T cells proliferate at certain locations within the organ (Dalum et al. 2016). It has been our aim to try to classify this tissue either as a primary or secondary lymphoid organ (Pabst 2007). The absence of recombinatory activities suggests that it cannot be a primary lymphoid organ. Functional experiments have revealed some stunning responses in the ILT: when bath challenged with the ISA virus, antigen was not located within the tissue, but a transcriptional response was observed, and most surprisingly, a physical reduction in the structure’s size was recorded (Austbø et al. 2014). The reason for this reduction is not apparent but could theoretically be related to T cell exodus from the ILT. No immune cell proliferation within the ILT was recorded in these experiments. Also, no antigen was discovered within the ILT. Normally, one should expect a secondary lymphoid organ to contain antigen and respond accordingly following exposure. Indeed, the hallmark of mammalian MALTs are their ability to sample exogenous antigens directly from the mucosal surfaces (Smith et al. 2013), but with no such reports so far published, this seems as an unlikely possibility with respect to the ILT. Combined with the observations of a surface towards the environment consisting of flattened epithelial cells with some resemblance to stratified squamous epithelium, but containing numerous goblet cells (Fig. 5) (Haugarvoll et al. 2008; Koppang et al. 2010), the conditions for antigen sampling seem poor. With such properties, the epithelium cannot be compared with the dome epithelium so important for antigen sampling in traditional MALTs (Smith et al. 2013). However, several studies in addition to those already mentioned have addressed responses and expression analyses of the ILT (Austbø et al. 2014; Aas et al. 2014, 2017; Kato et al. 2018), but so far, the ILT not only appears as an organ with unique anatomical features compared with other lymphoid tissues, but also as a structure with unique functions, of which we so far only understand a fraction.

## The salmonid bursa

**Fig. 6:**
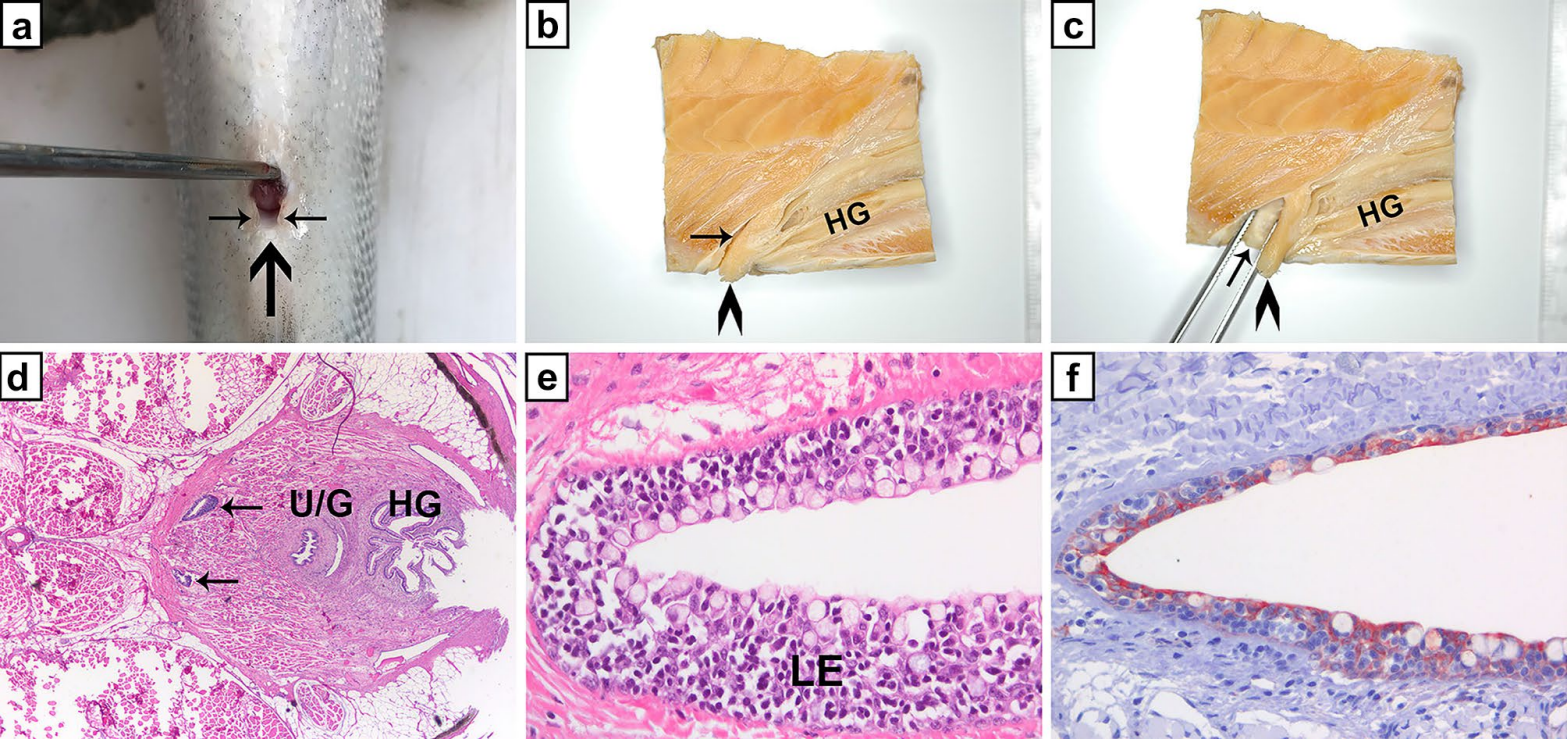
The salmonid bursa, Atlantic salmon. **a** Macroscopic location of the bursa caudal to the urogenital papilla (large arrow). Note the anal labiae (small arrows) which may be closed *in vivo*. **b** Macroscopic observation of the salmonid bursa, sagittal section. Bursa (arrow), the orifices of the urogenital papilla (arrowhead) and hindgut (HG) are all marked. **c** The bursa (arrow) may be opened with forceps for a closer inspection. Note the whitish epithelium which may be indicative for a high content of leukocytes. The urogenital papilla (arrowhead) and hind gut (HG) are marked. **d** Micrograph, transverse section, HE stained. The bursa terminates in two sacks with prominent lymphoepithelium (arrows). The ducts of the urogenital papilla (U/G) and the hindgut (HG) are seen in the section. **e** Micrograph, transverse section, HE stained, mid part of the salmonid bursa. Note the prominent lymphoepithelium (LE). **f** Micrograph, transverse section, cytokeratin immunostain, mid part of the salmonid bursa. Note the epithelial meshwork (red) in which lymphocytes are embedded

In 2019, our group discovered a hitherto non-reported structure in the anal region of the Atlantic salmon. Originally, we were searching for immune tissues in the abdominal channels connecting the abdominal cavity with the external milieu (George et al. 1982). However, these investigations promoted the identification of the “salmonid bursa” (Fig. 6) (Løken et al. 2020). This structure appeared to be a mucosal bursa with topographical similarities to the avian bursa of Fabricius. The investigation of the salmonid bursa is in its infancy. Of importance, our results so far show that the bursa develops a thick and prominent lymphoepithelium dominated by T cells. The epithelium involutes at sexual maturation and appears then as scale-free truncal skin. Thus, it distinguishes itself from the ILT which only appears to increase in size following body growth. The salmonid bursa develops from ectodermal invaginations caudal to the urogenital apertures just like in birds. The expression of CCL19 seems to be a driving force in the formation of the lymphoepithelium. Just like the ILT, no vasculature seems present in its thick lymphoepithelium, and the innervation is unknown. And just like the situation for the ILT, the bursal lymphoid tissue is confined to the epithelial compartment and does not seem to involve the lamina propria beneath the basal membrane. Such a structure is very different from avian and mammalian mucosa-associated organs where the lamina propria forms the main immune compartment (Smith et al. 2013). However, it is noteworthy that in mammals, the intraepithelial T cells are mainly CD8-positive T cells, but also CD4-positive T cells are present (Smith et al. 2013; Konijnenburg and Mucida 2017). α/β T cells were much more abundant in the salmonid bursa lymphoepithelium compared with the ϒ/δ population, and here, also abundant IgD-positive cells were present. IgM-positive cells were not as numerous, and the amount of IgT-positive cells was negligible (Løken et al. 2020). Clearly, this epithelium displays several surprises. Not only is the amount of Ig-positive cells surprising, but the lack of IgT-positive cells is stunning, as IgT has been shown to be important in mucosal immunity. With its location in the cloacal region of the salmon, the bursa is probably highly exposed to both enteric and environmental antigens and the need for local immune defence systems seem obvious.

Presently, we have no published information of any similar structures in other fish species, although we have observed a similar structure in brown trout (*Salmo trutta*) (E.O.Koppang, unpublished).

## Nasopharynx-associated lymphoid tissue

**Fig. 7:**
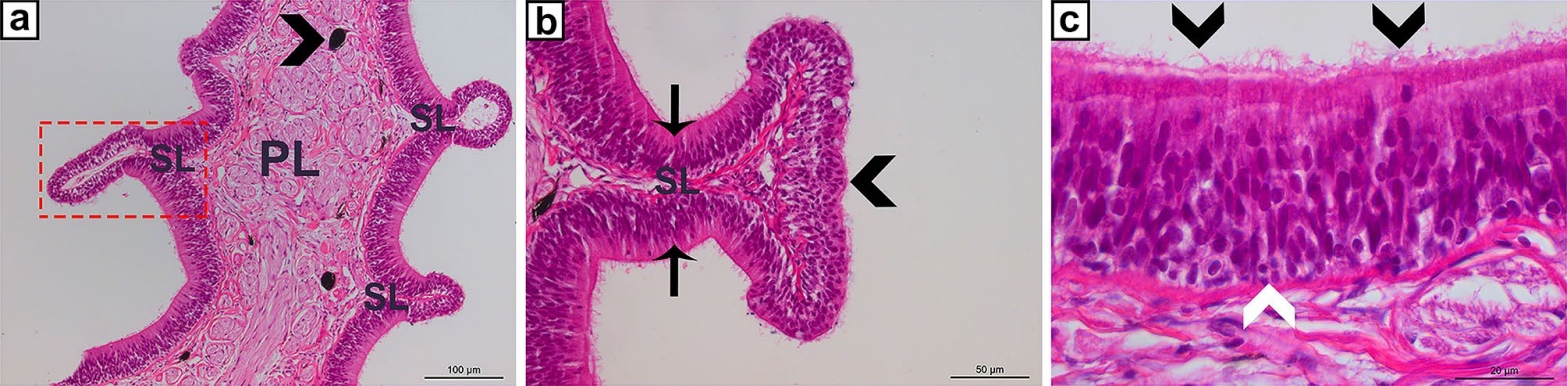
Sections from the olfactory organ, Atlantic salmon, HE stained. **a** A primary lamella (PL) with secondary lamellae (SL and boxed). Note that the primary lamella is filled with nervous tissue and abundant melanin-containing cells (arrowhead). **b** Enlarged image of a secondary lamella showing the two epithelial compartments; tips (arrowhead) and olfactory epithelium (arrows). **c** In the olfactory epithelium, lymphocytes may be observed (white arrowhead). Note the cilia on the epithelial cells (black arrowheads)

The teleost NALT can hardly be regarded as a lymphoid organ in its own right, but it seems nevertheless appropriate to mention it in this review of fish immune organs, as distinct regions containing immune cells can be identified. A comprehensive review of this tissue has just recently been published (Das and Salinas 2020) and we will only briefly present main features here as outlined by the mentioned authors. The olfactory organ of teleosts harbours two distinct mucosal compartments, i.e. “tips” and olfactory epithelium, both with a variety of different immune cells (Fig. 7). Both local and systemic responses to nasal immunization and infections have been observed in teleost fish (Magadan et al. 2019). The olfactory organ is innervated by autonomic nervous fibres and the prospects for studies of the interactions between the nervous and immune systems in this region are eminent. Of note, the African lungfish, although not a teleost, NALT structures, fulfilling the criteria of a lymphoid tissue, have been identified (Tacchi et al. 2014).

## Conclusions

If we wish to understand the actions of genes and their proteins, this information must be put into an anatomical and functional context. Any functional context can first be appreciated and understood with the recognition of the structures in which these functions take place. The recent discoveries of seemingly major lymphoid organs in fish suggest that we still have a long way to go in our understanding of the teleost immune system. But with developing technical improvements with respect to morphological approaches combined with gene expression studies will greatly improve our possibilities in revealing new aspects of structure and function of the fish immune system. This will finally enhance our understanding of the evolution of the immune system, in addition to providing a better fundament for disease control within the aquaculture industry.

## References

[CR1] Aas IB (2014). Transcriptional characterization of the T cell population within the salmonid interbranchial lymphoid tissue. J Immunol.

[CR2] Aas IB (2017). The interbranchial lymphoid tissue likely contributes to immune tolerance and defense in the gills of Atlantic salmon. Dev Comp Immunol.

[CR3] Abelli L et al (1998) Apoptosis of thymocytes in developing sea bass *Dicentrarchus labrax* (L.). Fish Shellfish Immunol 8(1):13–24

[CR4] Agius C, Roberts R (2003). Melano-macrophage centres and their role in fish pathology. J Fish Dis.

[CR5] Austbø L (2014). Transcriptional response of immune genes in gills and the interbranchial lymphoid tissue of Atlantic salmon challenged with infectious salmon anaemia virus. Dev Comp Immunol.

[CR6] Bajoghli B (2011). A thymus candidate in lampreys. Nature.

[CR7] Bjørgen H et al (2019a) Visualization of CCL19-like transcripts in the ILT, thymus and head kidney of Atlantic salmon (*Salmo salar* L.). Fish Shellfish Immunol 93:763–765 10.1016/j.fsi.2019.08.044 31422180

[CR8] Bjørgen H et al (2019b) Melanized focal changes in skeletal muscle in farmed Atlantic salmon after natural infection with *Piscine orthoreovirus* (PRV). J Fish Dis 42(6):935–945 10.1111/jfd.12995 PMC6850454 30972792

[CR9] Bjørgen H (2020). Anatomy, immunology, digestive physiology and microbiota of the salmonid intestine: Knowns and unknowns under the impact of an expanding industrialized production. Fish Shellfish Immunol.

[CR10] Bowden T, Cook P, Rombout J (2005). Development and function of the thymus in teleosts. Fish Shellfish Immunol.

[CR11] Castillo A (1990). Enzyme-and immuno-histochemical study of the thymic stroma in the rainbow trout, Salmo gairdneri. Richardson Thymus.

[CR12] Castillo A (1998). Thymic barriers to antigen entry during the post-hatching development of the thymus of rainbow trout. Oncorhynchus mykiss Fish Shellfish Immunol.

[CR13] Chilmonczyk S (1983). The thymus of the rainbow trout (Salmo gairdneri) light and electron microscopic study. Dev Comp Immunol.

[CR14] Chilmonczyk S (1992). The thymus in fish: Development and possible function in the immune response. Annu Rev Fish Dis.

[CR15] Dalum AS (2015). The interbranchial lymphoid tissue of Atlantic Salmon (*Salmo salar* L) extends as a diffuse mucosal lymphoid tissue throughout the trailing edge of the gill filament. J Morphol.

[CR16] Dalum AS (2016). Morphological and functional development of the interbranchial lymphoid tissue (ILT) in Atlantic salmon (*Salmo salar* L). Fish Shellfish Immunol.

[CR17] Das PK, Salinas I (2020) Fish nasal immunity: From mucosal vaccines to neuroimmunology. In: Fish Mucosal Immunology (special issue). Fish Shellfish Immunol 104;165-171 10.1016/j.fsi.2020.05.076 32497724

[CR18] Demoll R, Harder W (1964) Handbuch der binnenfischerei mitteleuropas Vol 2A, Anatomie der Fische. E. Schweizerbart'sche Verlagsbuchhandlung

[CR19] Di Lorenzo M (2020). Adrenal gland response to endocrine disrupting chemicals in fishes, amphibians and reptiles: A comparative overview. Gen Comp Endocrinol.

[CR20] Espenes A (1995). Immune-complex trapping in the splenic ellipsoids of rainbow trout (Oncorhynchus mykiss). Cell Tissue Res.

[CR21] Espenes A (1995). Investigation of the structural and functional features of splenic ellipsoids in rainbow trout (Oncorhynchus mykiss). Cell Tissue Res.

[CR22] Fänge R, Nilsson S (1985). The fish spleen: structure and function. Experientia.

[CR23] Fernandes CE et al (2019) Kidney anatomy, histology and histometric traits associated to renosomatic index in *Gymnotus inaequilabiatus *(Gymnotiformes: Gymnotidae)*.* Neotrop Ichthyol 17(4)

[CR24] Fischer U (2005). The ontogeny of MHC class I expression in rainbow trout (Oncorhynchus mykiss). Fish Shellfish Immunol.

[CR25] Flajnik MF (2018). A cold-blooded view of adaptive immunity. Nature Rev Immunol.

[CR26] Gallo VP, Civinini A (2003) Survey of the adrenal homolog in teleosts*. *In: International Review of Cytology. Kwang, WJ (ed.). Elsevier, pp 89–187 10.1016/s0074-7696(03)30003-8 14692682

[CR27] George C, Ellis A, Bruno D (1982). On remembrance of the abdominal pores in rainbow trout, Salmo gairdneri Richardson, and some other salmonid spp. J Fish Biol.

[CR28] Geven EJ, Klaren PH (2017). The teleost head kidney: Integrating thyroid and immune signalling. Dev Comp Immunol.

[CR29] Gillis JA, Tidswell OR (2017). The origin of vertebrate gills. Curr Biol.

[CR30] Greenwood MP (2009). The corpuscles of Stannius, calcium-sensing receptor, and stanniocalcin: Responses to calcimimetics and physiological challenges. Endocrinol.

[CR31] Harder W, Sokoloff S (1976). Anatomie der Fische.

[CR32] Haugarvoll E (2006). Melanogenesis and evidence for melanosome transport to the plasma membrane in a CD83+ teleost leukocyte cell line. Pigment Cell Res.

[CR33] Haugarvoll E (2008). Identification and characterization of a novel intraepithelial lymphoid tissue in the gills of Atlantic salmon. J Anat.

[CR34] Hellberg H (2013). Mast cells in common wolffish *Anarhichas lupus* L.: ontogeny, distribution and association with lymphatic vessels. Fish Shellfish Immunol.

[CR35] Huttenhuis HB (2005). Rag expression identifies B and T cell lymphopoietic tissues during the development of common carp (Cyprinus carpio). Dev Comp Immunol.

[CR36] Hyttel P et al (2009) Essentials of domestic animal embryology. WB Sauders Co Ltd.

[CR37] Kato G (2018). A novel antigen-sampling cell in the teleost gill epithelium with the potential for direct antigen presentation in mucosal tissue. Front Immunol.

[CR38] Koppang E et al (1998a) Differing levels of Mhc class II β chain expression in a range of tissues from vaccinated and non-vaccinated Atlantic salmon (*Salmo salar* L.). Fish Shellfish Immunol 8(3):183–196

[CR39] Koppang E et al (1998b) Expression of Mhc class I mRNA in tissues from vaccinated and non-vaccinated Atlantic salmon (*Salmo salar* L). Fish Shellfish Immunol 8(8):577–587

[CR40] Koppang E (2003). Production of rabbit antisera against recombinant MHC class II β chain and identification of immunoreactive cells in Atlantic salmon (*Salmo salar*). Fish Shellfish Immunol.

[CR41] Koppang EO (2010). Salmonid T cells assemble in the thymus, spleen and in novel interbranchial lymphoid tissue. J Anat.

[CR42] Lam S (2002). Morphologic transformation of the thymus in developing zebrafish. Developmental dynamics: An official publication of the American Association of Anatomists.

[CR43] Larsen HA (2013). Transcription of the tyrosinase gene family in an Atlantic salmon leukocyte cell line (SHK-1) is influenced by temperature, but not by virus infection or bacterin stimulation. Dev Comp Immunol.

[CR44] Løken OM (2020). A teleost structural analogue to the avian bursa of Fabricius. J Anat.

[CR45] Magadan S (2019). Nasal vaccination drives modifications of nasal and systemic antibody repertoires in rainbow trout. J Immunol.

[CR46] Matsunaga T, Rahman A (2001). In search of the origin of the thymus: The thymus and GALT may be evolutionarily related. Scand J Immunol.

[CR47] Mohammad M (2007). Anatomy and cytology of the thymus in juvenile Australian lungfish. Neoceratodus forsteri J Anat.

[CR48] Noga EJ (2006) Spleen, thymus, reticulo-endothelial system, blood. In: Ferguson HW (ed) Systemic pathology of fish. Scotian Press, London, A text and atlas of normal tisues in teleosts and their responses in disease, pp 121–139

[CR49] Pabst R (2007). Plasticity and heterogeneity of lymphoid organs: What are the criteria to call a lymphoid organ primary, secondary or tertiary?. Immunol Lett.

[CR50] Paiola M et al (2017) Oestrogen receptor distribution related to functional thymus anatomy of the European see bass *Dicentrarchus labrax*. Dev Comp Immunol 77:106–120 10.1016/j.dci.2017.07.023 28756001

[CR51] Pancer Z (2004). Somatic diversification of variable lymphocyte receptors in the agnathan sea lamprey. Nature.

[CR52] Picchietti S (2015). MHC II-β chain gene expression studies define the regional organization of the thymus in the developing bony fish *Dicentrarchus labrax* (L.). Fish Shellfish Immunol.

[CR53] Press CM, Evensen Ø (1999). The morphology of the immune system in teleost fishes. Fish Shellfish Immunol.

[CR54] Rességuier J (2020). Lymphoid tissue in teleost gills: variations on a theme. Biology.

[CR55] Rombout J (2005). Phylogeny and ontogeny of fish leucocytes. Fish Shellfish Immunol.

[CR56] Salinas I and Miller RD (2015) Comparative phylogeny of the mucosa-associated lymphoid tissue. In: Mestecky J, Strober W, Russell M, Cheroutre H, Lambrecht BN, Kelsall B (eds) Mucosal Immunology. Elsevier, pp 145–159

[CR57] Sichel G, Scalia M, Mondio F, Corsaro C (1997) The amphibian Kupffer cells build and demolish melanosomes: an ultrastructural point of view. Pigment Cell Res 10(5):271–287. 10.1111/j.1600-0749.1997.tb00687.x 9359623

[CR58] Smith P, Mcdonald T, Blumberg R (2013). Principles of Mucosal Immunology.

[CR59] Studdert V, Gay C, Blood D (2012). Comprehensive Veterinary Dictionary.

[CR60] Tacchi L (2014). Nasal immunity is an ancient arm of the mucosal immune system of vertebrates. Nat Commun.

[CR61] Takeuchi T, Gonda T (2004). Distribution of the pores of epithelial basement membrane in the rat small intestine. J Vet Med Sci.

[CR62] Thorsen J, Høyheim B, Koppang EO (2006). Isolation of the Atlantic salmon tyrosinase gene family reveals heterogeneous transcripts in a leukocyte cell line. Pigment Cell Res.

[CR63] van Konijnenburg DPH, Mucida D (2017). Intraepithelial lymphocytes. Curr Biol.

[CR64] Varga I (2008). The phylogenesis and ontogenesis of the human pharyngeal region focused on the thymus, parathyroid, and thyroid glands. Neuroendocrinol Lett.

[CR65] Vogel W (2010). Zebrafish and lymphangiogenesis: a reply. Anat Sci Int.

[CR66] Wang X (2014). Recombination-activating gene 1 and 2 (RAG1 and RAG2) in flounder (Paralichthys olivaceus). J Biosci.

[CR67] Zapata A, Amemiya C (2000) Phylogeny of lower vertebrates and their immunological structures. In: Origin and evolution of the vertebrate immune system. Springer, pp 67–107 10.1007/978-3-642-59674-2_5 10793475

[CR68] Zwollo P (2005). B cell heterogeneity in the teleost kidney: Evidence for a maturation gradient from anterior to posterior kidney. J Immunol.

